# Nature play in early childhood education: A systematic review and meta ethnography of qualitative research

**DOI:** 10.3389/fpsyg.2022.995164

**Published:** 2022-11-10

**Authors:** Jannette Prins, Femke van der Wilt, Chiel van der Veen, Dieuwke Hovinga

**Affiliations:** ^1^Department of Education, Thomas More University of Applied Sciences, Rotterdam, Netherlands; ^2^Department of Educational and Family Studies, Faculty of Behavioural and Movement Sciences, Vrije Universiteit Amsterdam, Amsterdam, Netherlands; ^3^LEARN! Research Institute, Faculty of Behavioural and Movement Sciences, Vrije Universiteit Amsterdam, Amsterdam, Netherlands; ^4^Department of Education, University of Applied Sciences Leiden, Leiden, Netherlands

**Keywords:** play, nature-based environment, play environment, early childhood education, nature play, cognitive development

## Abstract

Play in nature-based environments in childhood education has positive benefits for child development. Although previous reviews showed the benefits of play in nature-based environments for child development they did not attempt to understand how and why nature-based environments contribute to play quality. This review aims to explore the value of play in nature-based environments compared to non-nature-based environments for developmental outcomes of young children (2–8 year). We searched for studies that investigated the relation between play and nature-based environments on the databases PsycINFO, ERIC, and Web of Science. Inclusion/exclusion criteria were: (1) the study focused on play in/on a nature based environment, (2) the study included participants between the age of 2–8 years, (3) it was an empirical study, (4) the study was conducted in the context of early childhood education (ECE), and (5) the study included participants without special needs or disabilities. Using these criteria we selected 28 qualitative studies with an overall sample size of *N* = 998 children aged 2–8 years. The studies were synthesized using an adaptation of Noblit and Hare’s meta-ethnographic approach. Three overarching themes were found: (1) the aspects of play quality that are related to nature-based environments, (2) the aspects of nature-based environments that support play, and (3) the aspects of teacher-child interactions that contribute to nature play quality. The meta themes resonate with play theories and theories of the restorative value of nature. We draw on the qualitative data to refine and extend these theories, and to come up with a definition of the concept “nature play.” This systematic review also sets a base for future research on play interventions in nature-based environments. We argue that (1) research will benefit from thoroughly conceptualizing the role of play in the development of young children, (2) using the affordances theory research will benefit from moving beyond the individual play actions as a unit of analysis, and (3) from an educational perspective it is important to shift the focus of nature play to its benefits for children’s cognitive development.

## Introduction

In early childhood education (ECE), play and learning are inextricably intertwined (Hirsh-Pasek, 2008). Play is often considered as a context for young children’s learning and development, and can take place indoors (e.g., in a classroom) as well as outdoors (e.g., in a nature-based environment). However, outdoor play in ECE is often done for its value to relax and recover from the important play and learning time that takes place indoors. As a result, in ECE play in outdoor settings is not often valued for its potential benefits for children’s learning development (Miranda et al., 2017). Recently, many studies have focused on play and learning in nature-based environments. Based on these studies, this review aims to explore the value of play in nature based environments in ECE. The research for this review was guided by the following question: what is the value of play in nature-based environments compared to non-nature-based environments for developmental outcomes of young children (2–8 year).

### Play as a context for child development, three perspectives

In most cultural communities, play is a major aspect of children’s life (Roopnarine, 2012). Most play researchers agree on the importance of play in early childhood. In fact, play is seen as a key element of child development because it is the context for the development of cognition (including language), motor skills and social-emotional competence (Rubin et al., 1983; Golinkoff et al., 2006; Nathan and Pellegrini, 2010).

To affirm the importance of play, in Article 31 of the United Nations Convention on the Rights of the Child (United Nations, 1989) play is viewed as a fundamental need and right of children. This need for and right to play needs to be respected in the lives of young children. Consequently, article 31 challenges us to understand play from the perspective of children’s needs and rights.

Before play ended up as a fundamental right in the Children’s Rights Treaty, the critical role of play has been studied by many scholars using different theoretical frameworks. According to Wynberg et al. (2022), roughly three theoretical perspectives can be distinguished. First, Piaget describes in *Play, Dreams and Imitation in Childhood* (Piaget, 2013), how children incorporate objects and events of the world around them in their play, creating a mental model of the world. In this genetic epistemology perspective, children’s level of cognitive development is reflected in types of play (functional and constructive play, symbolic/fantasy play and games with rules). Piaget’s theory of cognitive development suggests four phases in which intelligence changes as children grow. For early childhood the first three are relevant: children (0–12 year) grow from sensorimotor intelligence (e.g., children understand the external world only by sensing and touching objects that are present), into preoperational intelligence (e.g., during this period children are thinking at a symbolic level but are not yet using cognitive operations, they still need to act in the external world to perform these operations) into concrete operational intelligence (e.g., children can use logic and transform, combine and separate concepts on a mental level) In this way, children’s play can be classified on the basis of their cognitive development, but children’s play is not seen as a context for new development. Therefore, this theoretical perspective does not explain how children’s play quality and the physical environment are related.

Secondly, in contrast to Piaget’s view that play reflects the actual level of children’s cognitive development, in Vygotsky’s cultural historical activity theory (CHAT), play is considered a social activity in which children meet and interact with the social cultural environment. With help of parents, educators and peers, children gain in play a driving force for further cognitive, social-emotional, and motor development (Nicolopoulou, 1993).

Leontiev advanced Vygotsky’s theory by differentiating play actions from play activity. Play actions are performed to achieve a single goal. A play activity is a set of related play actions that meet children’s need to get to know the world around them and be able to contribute to it. Their play activity derives its meaning from the satisfaction of fulfilling this need, which is the motive for their activity. However, the goal of a play action does not necessary coincide with the motive of the activity. In fact, the single goal of an action often comes apart from this motive. For instance, children in a nature-based environment collect sticks (action) to build a pretend bonfire (activity) to fulfill their need to get the feel of making a bonfire (not because they were cold or needed to cook).

Within CHAT, tool use is an important aspect of play activity. Tools help children to fulfill their need and these (symbolic) tools link the action (collecting sticks) to their motive (getting to know bonfires by pretending to make one). In other words, children are motivated by these tools. In the play context, tools have agency to achieve goals (Bodrova and Leong, 2015; Wynberg et al., 2022) and motivation to use the tools is what makes children act, think and develop (Nicolopoulou, 1993; Deci and Ryan, 2008; Bakhurst, 2009). As a result of engaging in play, the perceptual world–i.e., the world the child meets through perceptually interacting with it–becomes a conceptual world of meaning and value. In this process, the child develops the mental power to understand the (meaning of) the world that surrounds him/her. The perceptual world invites or affords play activity (Bakhurst, 2009). In the example of children building a bonfire, the sticks mediate between the perceptual and conceptual world, children use their mental power to imagine the real fire and the heat that comes from it, while building the bonfire and gathering around it. Although CHAT accounts for the role of the physical environment in children’s play, the environment is mostly viewed as situated in a socio-cultural environment.

Thirdly, Gopnik (2020) describes childhood from an evolutionary perspective as a time for the human mind to explore the unpredictable range of human possibilities. To develop the capacity to navigate the perceptual world, in other words to get the feel or hang of it, children actually have to feel the world and hang around in it. During childhood, children are especially prone to explorative and “active” learning. While involved in messy and intuitive play actions, children gather new information about the world around them, learning and adapting without using adult intelligence, such as planning or focused attention. Instead, they get involved with all their senses to imagine even far-away and unlikely hypotheses, such as using objects during play in a creative way, not being hindered by experience of the usual function of the object (Gopnik and Wellman, 2012; Schulz, 2012; Wente et al., 2019). Within the evolutionary perspective childhood is an extended time for exploration of an environment that is variable, with a mix of predictability and unpredictability. In the same way as the CHAT, within the evolutionary perspective the focus is on cultural learning, i.e., obtaining information from other humans and not so much from the interaction with the nature-based environment.

Although these three perspectives differ in focus and methodology, they all acknowledge play as important for child development. During play children find out the meaning of the world that surrounds them, including the physical world, and learn how they can interact with it. In this way they develop as human beings with cognitive, social, emotional, and motor competencies.

### Defining play

In this review, we focus on play and how the quality of play might be supported by the physical environment where children play. Therefore, we need a definition to distinguish play behavior from other behavior. As we have seen in the literature on play there is no defining key factor that connects all actions that are recognized as play actions. In *the Oxford handbook of the development of play*, Burghardt (2012) comes up with a set of five criteria that characterize the play of all animals: (1) It is not fully functional in the form in which it is expressed; play actions can look functional but the actions do not contribute to survival; (2) It is spontaneous, voluntary, intentional, pleasurable, and done for the sake of playing; (3) Play differs from functional behavior in structure or timing in at least one respect: incomplete, awkward, and precocious; (4) It is performed repeatedly but not in a stereotyped way; and (5) It is initiated when the animal is “relaxed”: well fed, warm and safe. These five criteria partly overlap with the dispositions described by Rubin et al. (1983). They define play as: (1) intrinsically motivated; (2) for the sake of play(ing); (3) deriving pleasure from it, and; (4) having the freedom to modify the rules within the play (Rubin et al., 1983). For this review, we will combine the aforementioned criteria and include all behaviors that can be classified as a child’s interaction with the environment, while being highly involved, intrinsically motivated, deriving pleasure from it, and having the freedom to modify the rules (cf., Rubin et al., 1983).

### The quality of the physical environment in relation to play quality

The physical environment where children play is part of their play. The value of explorative and active play is directly related to both the complexity of the physical environment and the opportunity to incorporate the environment in play (Gopnik, 2020). In other words, an environment not only serves as a play décor, but it also serves as a place that affords play. For example, findings from systematic reviews consistently demonstrate that a nature-based environment affords different play behavior compared to non-nature-based environments (Gill, 2014; Dankiw et al., 2020; Zare Sakhvidi et al., 2022). How can this be explained?

The affordances theory of Gibson (2014) is a way to describe an environment in terms of the distinctive features that offer possibilities for play behavior for a child or a group of children. An affordance is something that refers to both the environment and the skills of a child at that moment. The affordance theory helps to understand why nature-based environments differ from non-nature-based environments. For instance, a tree can afford leaning for a 1-year old, hiding for a 5-year old and climbing for a 7-year old. Heft (1988) and Kyttä (2002) advanced the affordances theory into a functional taxonomy, by describing the distinctive functional properties of an environment, properties that are both objectively real and psychological relevant. It is a way to describe the setting, the person (the child with her skills at that moment) and the action as a “system.” According to Heft (1988), the functional possibilities for meaningful play that children perceive in nature-based environments are different from the possibilities they perceive in non-nature-based environments.

In addition to the affordances theory to describe the assets of nature-based environments for play, two complementary theories from research on nature-based environments are related to aspects of play (quality) as well: the Stress Recovery Theory (SRT) and the Attention Restoration Theory (ART) (Ulrich, 1983; Kaplan, 1995; Berto, 2014). SRT is a psycho-evolutionary theory that states that since humans evolved over a long period in natural environments, people are to some extent physiologically and perhaps psychologically better adapted to nature-based environments as to non-nature based environments. ART is a psycho-functionalist theory that states that humans have an innate predisposition to pay attention and respond positively to natural content (e.g., vegetation and water) and to settings that helped survival during evolution. Both theories state that nature-based-environments are more restorative than non-nature-based environments; according to SRT, nature-based environments relieve physiological stress whereas according to ART, nature-based environments restore mental fatigue. In this way nature-based environments contribute to play quality as we look at the criteria for play quality mentioned above: a child can only initiate play when it is relaxed, and play asks for involvement and attention.

### Defining nature-based environments

As we see how the quality of the play activity of a child is intrinsically linked to the nature-based environment, we need a definition to distinguish a nature-based environment from other environments. As it is difficult to find one key factor to define play, there is also no such key factor that connects all environments recognized as nature-based environments. To describe such an environment the affordances theory of Greeno (1994), Gibson (2014), and Lerstrup and Konijnendijk van den Bosch (2017) makes it possible to look at an environment in terms of affordances. He described five affording features of an environment: (1) places, (2) attached and (3) detached objects, (4) substances, and (5) events. In this review, we use these features to distinguish nature-based environments from non-nature-based environments. Nature-based environments (1) have a surface (place) that is the basis for growth of living elements, (2) provide possibilities for interacting with living, non-man-made elements like plants, trees, and insects, (3) these living elements “provide” loose materials to play with, such as sticks, seeds, feathers, and shells (attached and detached “objects”), (4) non-living elements are part of a nature-based environment as these elements are connected to the biosphere of the living elements such as water, rocks, and soil (substances), and (5) weather elements such as fresh air, rain, wind and sunshine, or seasonal elements such as blooming or decay are the features that ensure change (events) (Gill, 2014; Chawla, 2015; Dankiw et al., 2020).

### The role of the teacher

For this review, we also investigated the role the teacher has in designing and/or choosing the play environment. The motivation and the capacity to be taught by the world is not totally innate. It needs to be nurtured and sustained by adults. Early childhood teachers are part of the play context and have a role in mediating between the child and the world. In this context they also have a role in the acquisition and use of language during play. While the perceptual world with its structure and rules becomes a conceptual world in play the acquisition and use of language makes it possible to store the concepts in the mind (Huizinga, 2014). Most play theories agree on the role early childhood teachers have in guarding children’s play, enriching children’s play environment, and protecting children for dangers, but there is considerable debate on the question if and how adults should participate in children’s play activities (van Oers, 2013).

### Reason for this review

Reasoning from play theories and the environmental psychologist theories we might expect that nature-based play environments, as an indivisible part of children’s play actions, can contribute to children’s cognitive, social-emotional, and motor development.

In the last decade, many studies have been conducted into the relation between a healthy development of children and engagement in nature-based environments. Most of these studies have focused on health and physical activity. The reviews of Gill (2014), Chawla (2015), and more recently Dankiw et al. (2020) have provided overviews of the benefits of nature for children’s development. These reviews were focused on children between 1 and 12 years old. First, the systematic review of Gill showed the benefits of children’s engagement with nature on mental health as well as physical activity. Second, Chawla’s work was not so much a systematic review but a thorough reflection on research into the benefits of nature contact for children. She placed the research in the context of changing research approaches, thus showing how different research questions and methods shape our understanding of the benefits of access to nature for children. Third, Dankiw’s review investigated the impacts of children’s engagement with unstructured nature play, finding that unstructured nature play may have a positive impact on different aspects of child development. By focusing on developmental outcomes of quantitative studies, this study did not attempt to understand how or why unstructured nature play is related to these positive outcomes. A systematic review of qualitative studies can synthesize findings and advance the knowledge base of how nature-based environments contribute to play quality. Synthesizing the fragmented literature will contribute to a useful resource for guiding future research on this topic and inform early childhood educational practices, valuing nature-based play environments as intrinsically linked to play quality.

We systematically reviewed studies into play in nature-based environments in ECE. These studies may contribute to our understanding of the experiences of children and teachers in ECE when going outside to play in nature- based environments. Moreover, these experiences set out a basis for understanding the possibilities of playing in nature-based environments for cognitive, social-emotional, and motor development in ECE. We reviewed studies in early childhood educational settings since in these settings play is an important part of the curriculum.

## Methods

The Preferred Reporting Items for Systematic Reviews and Meta-Analysis (PRISMA) guidelines (Page et al., 2021) was adopted for the purposes of the present review. A PRISMA checklist is provided in Supplementary File 1.

### Inclusion and exclusion criteria

Articles were included if they met the following selection criteria:

(1) The study focused on play in/on a nature based environment (studies were excluded if the exposure to nature was not specified as “interaction” or “play” or if the environment where the children played did not match our criteria of nature based environments as stated in our introduction).
(2) The study included participants between the age of 2–8 years.
(3) It was an empirical study.
(4) The study was conducted in the context of ECE (studies were excluded if they were not conducted in a center for ECE, such as day care centers and preschools).
(5) The study included participants without special needs or disabilities.

### Databases and search query

Databases PsycINFO, ERIC, and Web of Science were used to identify studies that investigated the relation between play and nature-based environments. To ensure the quality of the studies we only included empirical studies that were published in peer-reviewed journals. Furthermore studies written in English that were published between May 1995 and 2022 were included. We combined keywords on the two major concepts of this review: play and nature-based environments. To ensure a comprehensive search the following keywords were used for play or activity: manipulative play, object play, relational play, block play, loose part play, outdoor play, free play, unstructured play, rough and tumble play, explorative play, creative play, construction play, physical play, gross motor play, role play, pretend play, social play, imaginative play, socio dramatic play, social pretend play, as if play or physical activity, unstructured activity, explorative activity, physical activity, construction activity, and gross motor activity. For the nature-based environment, the following keywords were used: green or natural environment, playground, landscape playscape setting area or space, school garden, school forest, school wetland, school wilderness, school grassland, greenery, garden, forest, wetland, wilderness, grassland, tree cover, tree canopy, biodiverse school ground, and nature based. Boolean operators were used to ensure that each possible combination of keywords was included. The search query is provided in Supplementary File 2.

### Selection procedure

**FIGURE 1:**
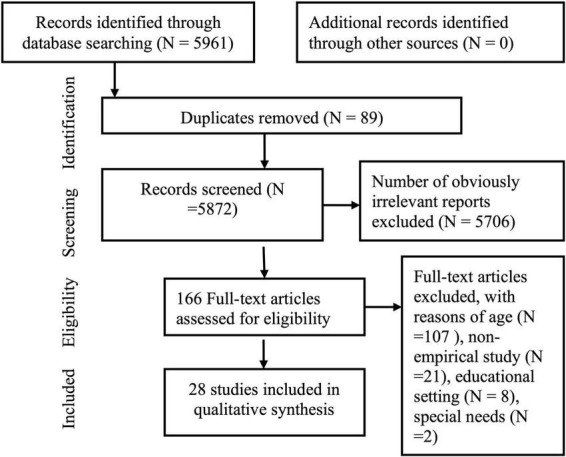
Study selection process.

The primary search resulted in a selection of 5,961 articles. Next, duplicates were removed, and titles, abstracts, and keywords of the remaining articles were manually screened. Many studies in this first selection were either in the field of environmental science or health, and did not concern playing children. After removing the studies that obviously did not meet our selection criteria we assessed 166 articles for eligibility. We excluded 107 studies for reasons of age. We also screened studies with participants between 2 and 8 years as well as participants beyond this age. We did not include them because it was impossible to decide if the results were specific for the group of children between 2 and 8 years. A random selection of twenty articles of the 166 articles were checked with two researchers, both members of a research group performing a systematic review in the field of ECE. They checked if the article met the criteria of our definition of play and nature based environment as stated in our introduction. Quality appraisal was made through the Joanna Briggs Institute (JBI) Critical Appraisal Tool for Qualitative Studies (Lockwood et al., 2020) (see Supplementary File 2). Using this tool we were surprised by the innovative and creative ways these studies adapted to respect the voice of young children. We ended up with a final selection of 28 studies with an overall sample size of *N* = 998 children aged 3–8 years. See Figure 1 for an overview of the study selection process.

### Data extraction and synthesis

The selected studies were analyzed and synthesized in four steps based on Noblit and Hare’s meta-ethnography method and adapted for this study (Agar, 1990; Noblit and Hare, 2012; Nye et al., 2016): Step 1: The studies were read and re-read to gain a detailed understanding of their theories and concepts and their findings according to the following categories: (1) Design/method, (2) theories and conceptualization, and (3) outcomes. Supplementary Table 1 gives an overview of the 28 studies, specified according to these categories. To retain the meaning of the primary concepts within individual studies and to define the relations between these concepts we developed codes regarding the experiences of children and teachers while playing in nature-based environments during ECE (i.e., authors’ interpretation of the data and “second order constructs”).

**FIGURE 2:**
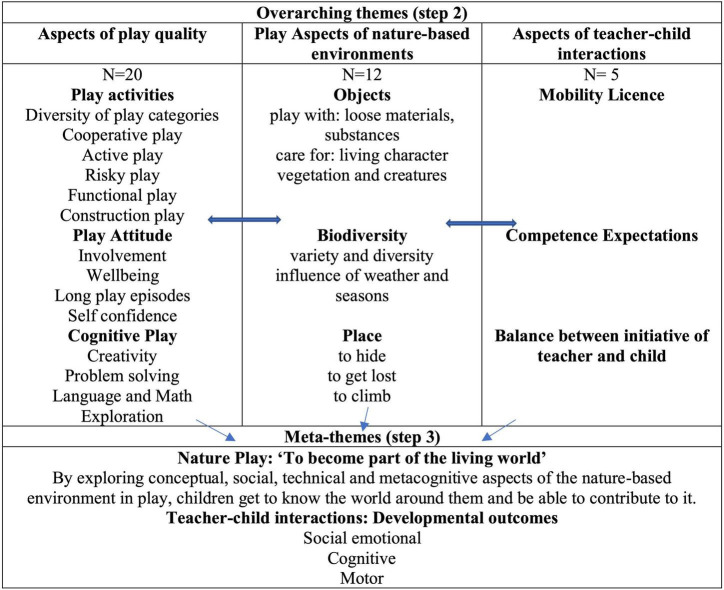
Meta-synthesis of key concepts into three themes and two Meta-themes.

Step 2: In order to determine how the studies were related, the initial codes were grouped according to key aspects of (1) play quality, (2) the nature-based environment, and (3) the teacher-child interactions. These key concepts from individual studies were synthesized, which resulted in lists of overarching themes for each of the three groups (see Figure 2).

Step 3: Studies were translated into one another to produce “meta-themes” across the different aspects of play in nature-based environments. To draw out the findings under each meta-theme, some studies were chosen as “index” papers from which we extracted findings. These index papers stood out in terms of their conceptual richness. Their findings were then compared to and contrasted with the findings of a second study, and the resulting synthesis of these two studies were then contrasted with a third study, and so forth. This is referred to as “reciprocal translation” (Noblit and Hare, 2012; Nye et al., 2016). For example Lerstrup and Konijnendijk van den Bosch (2017) advanced Gibsons and Hefts theory of affordances and functional classes of outdoor features into “key activities” afforded by classes of the outdoor environment. These new concepts were used for the translation of concepts from other papers that were related but not conceptualized in this way.

Step 4: The meta-themes from step 3 were synthesized according to aspects of quality of ECE. *Via* interpretive reading of these meta-themes we developed a “line of argument” synthesis regarding the value of play in nature-based environments for improving developmental outcomes of ECE. This is presented in the discussion.

## Results

### Meta method analysis

During step 1 we analyzed the study designs of the 28 included studies. The studies into play in nature-based environments in ECE all aimed to get more insight into the relation between children’s play and nature-based environments in ECE. The studies aimed to study a myriad of educational outcomes, such as physical activity, cognitive, social-emotional, and motor development as well as health. The relevance of these studies is motivated by concerns about changes in the practice of playing outside as healthy practice for young children’s physical and mental wellbeing. Opportunities for outdoor play have diminished drastically since the mid-20th century, due to cultural changes such as parental control and fear, inadequate access to outdoor playgrounds, screen time and the focus on cognitive development in ECE.

The studies included in the present review can all be characterized as small-scale studies using observations of play behavior in nature-based environments and interviews with teachers and children to explore their experiences of playing in nature-based environment. Participating early childhood settings in the studies were sampled based on their outdoor play practices including the design of their playgrounds. These studies can be divided into two groups: one that compared play on a nature-based (part of the) playground to play on (part of the) traditional designed playground and one that compared forest school practice to indoor/outdoor classroom practice.

In all studies, except for one, the sample size was given and ranged between *N* = 4 and *N* = 198, with a total of *N* = 998 and a mean of *N* = 36. Twelve of the studies had a sample size of <*N* = 20, 13 had a sample size between *N* = 20 and *N* = 100, one study had a sample size of *N* = 198, and one had a sample of teachers *N* = 63 teachers. One study did not specify the sample size. The relatively small sample sizes of most studies can be explained by the fact that the studies had an explorative and qualitative research design.

Seventeen studies used play observations describing different aspects of the relation between children’s play behavior and nature-based outdoor environments, to get more insight in how children use outdoor environments during outdoor play activities. In most studies these observations were characterized as phenomenological, ethnographical, and participatory. Blanchet-Cohen and Elliot (2011) for instance described how participatory observation was a primary method of listening to young children in unmediated ways to get insight in how the children used the nature based environment. In the studies of Moore et al. (2019) and Dyment and O’Connell (2013) observation was done by using event sampling or taking scans with an observation tool, making it easier to observe a higher number of participants.

In the studies where children’s views on their outdoor play experiences were explored, a mosaic approach was used to get insight into the views of young children, using arts-based data techniques while interviewing children. These studies were inventive and respected the way young participants are able to express their own views. For example, in the study of Streelasky (2019), drawings, paintings, and photographs were used during child interviews to support them in expressing their views. In the study of Moore et al. (2019), the children gave a tour around the yard to express their views on the value of the nature-based environment. Four studies also collected data from teachers, to explore their views and their interaction with children when playing outside in nature-based environments.

Although most studies used open observations to investigate the play activities of the children, some used validated instruments, such as the system for Observing Play and Leisure Activity in Youth (SOPLAY). This system is used by Fjørtoft (2001) as well as by Dyment and O’Connell (2013) and is a way to label children’s activities, for instance to assess the diversity of their activities, but it does not capture how these activities are related to the play environment. Another way to assess the quality of the play activities is in terms of involvement, freedom, and joy. In two studies, the Leuven Child Involvement Scale was used to analyse children’s play in terms of involvement and joy. Other studies (Luchs and Fikus, 2013, 2018; Morrissey et al., 2017) used the duration of the play episodes as a measure of the quality of the play: The longer children played, the higher the quality of their play episode.

In three studies instruments were used to assess the play potential of the nature-based outdoor environment. Mårtensson et al. (2009), for example, used the outdoor play environment categories (OPEC) tool, which gives a higher score to environments with large integrated spaces with plentiful greenery and varied topography compared to small areas where open spaces, play structures and vegetation are placed in separate parts of the environment. Richardson and Murray (2016) used the early childhood environment rating scale (ECERS) to assess the nature-based environment, but this tool is developed to assess indoor classrooms and is not adapted for outdoor spaces.

Four of the five studies that also used quantitative data, measured children’s physical activity in a quantitative way using accelerometers, and one study measured if features of the natural environment correlated with measures of inattentiveness.

Data analysis techniques were specified in all of the studies. In most of them (24 studies) comparative thematic analysis was used as data analysis technique. In the five mixed method studies, several statistical tests were used as well.

Details about strategies to address validity were not often mentioned, but four of the studies used focus groups of teachers to discuss the finding of the studies and to perform a member check.

### Meta concept and theory analysis

During step 2, we synthesized key concepts in the studies. The studies in this review were selected based on two conceptual criteria, one of them was the *nature-based environment*, the other concept was *play* (or aspects of play). Most studies used a specific theoretical framework and/or a philosophical perspective to explain and understand the expected relation between nature-based environments and play. These theories help us to conceptualize about and generalize the findings within the specific studies and help us to understand the limits of these generalizations.

#### Play

Seven studies used a specific theory in which the concept of play was embedded. Most of these studies used Vygotsky’s sociocultural theory, from which play can be defined as a mode of activity. However, the concept “activity” was mostly used as “the things children do” or, in other words, children’s actions. Certainly, the theory was not used to place play in the larger cultural-historical context. Other studies used a criterion- based definition of play, such as it was “free” or child initiated. For example, in the study of Brussoni et al. (2017) play was described in terms of activities chosen by the children. Different aspects of these activities in nature-based environments were explained, such as hierarchy between peers during play, the complexity of the play or the duration of play episodes. Other studies defined play as consisting of different play categories, some of them cognitively more complex. For example, in the study of Dyment and O’Connell (2013) play was described using five categories: functional, constructive, symbolic, self-focused, and talking, whereas the constructive and symbolic category was also coded as creative and imaginative. In the studies that focused on a specific type of play, such as physical play, risky play, or sociodramatic play, it was easier to extract the specific play concept. Morrissey et al. (2017) for instance, used a detailed description of the concept of sociodramatic play: involving two or more players, providing a crucial everyday context in which children are motivated to engage socially with peers, and practice skills in communication, negotiation, symbolic, and creative thinking.

#### Nature based environment

Twelve studies used Gibson’s affordances theory to distinguish nature-based environments from non-nature-based environments. Lerstrup and Konijnendijk van den Bosch (2017), for instance, used the affordances approach to operationalize how play actions are afforded by a specific feature of the environment and a specific user (a child of the preschool participating in their study) of that feature. In this way, the environment is not viewed as a separate object, but as something children take with them in their own experiences. Sandseter (2009) assessed how a nature-based environment affords risky play for pre-schoolers, using the concept of affordances, but adding the role of the educator to the equation.

Some studies used the concept “play opportunities” instead of affordances, to operationalize the relation between children’s play behavior and a nature-based environment. Canning (2013), for example, made observation notes of the play behavior during den-making sessions and focused on the conversations between children to explore how the environment offers opportunities for creative thinking. In the den-making context the nature-based environment is an integrated part of children’s play experience in the same way as the environment in the affordances approach. In short, in most of the studies the relation between nature-based and children’s play behavior is operationalized as observed activities afforded by nature-based outdoor environments.

Although all of the studies aimed to explore if and how (aspects of) children’s play behavior is afforded by nature-based outdoor environments, there is no generally accepted description of the concept “nature-based environment” and it is hardly operationalized in most of the studies. Fourteen studies (Supplementary Table 1 nrs. 2, 3, 6, 8, 11, 12, 14, 19, 20, 23, 24, 25, 27, and 28) used a comparator outdoor play environment to compare the nature-based environment with. The comparator environment that was referred to as “traditional” or “usual,” always contained man-made or manufactured elements such as a climbing structure and a sandpit. Another similarity in the description of elements that the non-nature-based environment consisted of was the character of the surface: it was paved, concrete, or hard. This is a kind of surface that afforded functional play: riding bikes, running around. These comparator environments can serve as a starting point to describe the (operationalized) characteristic elements of the nature-based environments in the studies.

In contrast, the elements of the nature-based environment were in the first place described as elements that were not man-made and do change, grow or die (even) without the intervention of humans. For instance, in the study of Brussoni et al. (2017) the “seven C’s system” for assessing the quality of the outdoor environment was used. One of the C’s stands for change: How does the play environment change over time? Second, although nature based environments can change, grow or die without human intervention, at the same time the elements of the nature-based environment are more sensitive to human intervention than man-made elements in an a non-nature based environment, for instance a climbing structure. Therefore, nature-based environments ask for care when playing with and in it, which interferes with the children’s play actions. Third, the surface of the nature-based environment is referred to as “biodiverse, soft, and diverse.” An example of this is the study of Puhakka et al. (2019). In this study, the greening of day-care yards consisted not only of adding green elements, but also of replacing the complete surface area of a day-care yard by forest floor, sod, peat blocks, and planters for vegetable growing, making the surface more biodiverse.

Related to the surface as an important element of the nature-based environment, in many studies natural loose parts found in or on this surface were a vital element of the nature-based environment affording specific play activities. Harwood and Collier (2017) even went a step further by not operationalizing the observed activities of the children afforded by nature-based outdoor environments, but by operationalizing the activities that the natural loose parts performed in the child’s play narrative. In this view, the agency of sticks in children’s multi modal texts was afforded by the children. This post-humanist perspective (as they called it) was interesting as it described how the agency of the children was enriched by focusing on the agency of the stick. To acknowledge the agency of nature-based environments might be a key factor in describing the special way it affords play, compared to other environments.

Three studies used a theory of place. These theories account for the fact that a child’s identity is nurtured and shaped by place (Gruenewald, 2003; Adams and Savahl, 2017; Crippen, 2017). Children have strong attachments to the places they play in and actively construct places for imaginative play (Hart, 1979).

### Meta data analysis

In step 3 we compared and contrasted the key concepts found in the studies to one another to establish overarching themes (reciprocal translation). Most of the studies showed that aspects of children’s play quality are related to aspects of nature-based environments which might lead to benefits for child development if mediated in certain ways by early childhood educators. However, this relationship is complex and it is not easy to isolate the elements of the physical environment from all other factors that influence play quality. In order to find how the outcomes of studies were related, we grouped the studies according to (1) aspects of play quality (2) aspects of nature-based environment, and (3) aspects of teacher-child interactions.

#### Theme 1: Aspects of play quality: play actions, play attitude, and cognitive play

All studies pointed out that there was a relation between children’s play actions and nature-based environments. Firstly, compared to a non-nature-based environment, there was more variety in play categories while children played in nature-based environments. In the studies, a non-nature-based environment mostly afforded a more physical type of play whereas nature-based environments afforded more diversity in type of play. For instance, Luchs and Fikus (2018) observed that children showed play patterns in which they combined different play types. Six studies reported more socio-dramatic play in the nature-based environment. In the study of Coates and Pimlott-Wilson (2019), for example, children reported that the forest site where they played offered them opportunities to make things and be creative, and enact their own stories.

Secondly, the vast majority of the studies reported how play in nature-based environments was related to children’s social-emotional attitude during play. Interesting were the studies that included children’s own perspectives on their play experiences in nature-based environments: Children often reported joy, wellbeing, and enthusiasm. For instance, in the study of Moore et al. (2019) they included “stories of agency” in which children demonstrated a strong sense of comfort and self-confidence with the nature-based environment, by telling about the freedom they felt to make footprints anywhere or to cool down in the grass. This sense of confidence was also found in the studies that observed more risky play in nature-based environments, or a higher degree of risk afforded by nature-based environment. In the study of Mcclain and Vandermaas-Peeler (2015), the degree of “wilderness” of the environment (a creek compared to a river) afforded the degree of challenge and risk in the observed play behavior. Some studies emphasized the possibility of the nature-based environment to sustain the play story, resulting in longer play episodes, compared to episodes on the non-nature-based playground. But also in using more play space, as the nature-based environment helped them to meander from one area to another. This relates to the studies that pointed to more explorative play behavior or higher involvement and engagement during play in nature-based environment. For example, McCree et al. (2018) found high scores of involvement during play sessions on a forest school site.

Thirdly, besides the fact that playing in a nature-based environment interacts with how children play in such an environment, five studies described how this is related to children’s cognitive development. In early childhood, cognitive development as an outcome of play activities is highly dependent on how much a child is involved in play and the extent to which the child experiences wellbeing. Seven studies observed explorative play behavior, problem solving and creativity and related this to the nature-based environment. For example, in the study of Puhakka et al. (2019), increasing biodiversity and the amount of greenery of school yards led to more explorative play, more multi-sensory play experiences, and better pre academic skills (i.e., counting) than before the intervention. In the longitudinal study of McCree et al. (2018) an improvement in academic attainment (i.e., reading, writing, and maths) was seen after 3 years of attending weekly forest school sessions compared to their non-participating peers at school. Richardson and Murray’s (2016) study was the only study that measured richer language use during forest school sessions, in terms of noun diversity, and the use of adjectives and verbs.

To summarize this step of reciprocal translation: when children play in nature-based environments, the quality of their experiences during play is improved. This is shown by a greater diversity in play actions while at the same time the duration of the play episode was extended, compared to their play in non-nature-based environments. Children’s involvement and wellbeing during play was intensified while playing in nature-based environments. Furthermore, they were not only physical active but also used different cognitive skills in their play.

#### Theme 2: Play aspects of nature-based environments

Although in theme 1 we showed that playing in nature-based environments relates to higher play quality, it was not yet connected to specific aspects of the nature-based environment. Theme 2 reveals that this higher play quality is connected to specific aspect of the nature-based environment. Most of the studies indicated a clear relation between nature-based environments and playing with loose or fixed natural materials. Playing with loose materials often leads to construction play. For instance, in the study of Puhakka et al. (2019) the researchers observed that children were doing more arts and crafts with the loose natural materials. In many other studies we reviewed, sticks were mentioned as natural materials with special interest. For instance, in Canning’s (2013) study children used sticks to lay out a ladder and to pretend to climb in it. In the study of Harwood and Collier (2017) the sticks even had agency, for instance they were friends carried and cared for by the child, being able to change the play narrative of the child. In four studies play with small creatures was mentioned (e.g., insects, worms, and snails), as well as care for plants and vegetation. These studies also pointed to the importance of the notion of abundance of natural materials as opposed to the notion of scarcity (for example of toys) in non-nature-based environments. Zamani (2013) described how the living character of nature-based zones sparked curiosity and wonder, and invited play with critters and plants. Also in the study of Wight et al. (2015) the fact that nature “lives” made children caring for it. In three studies the notion of place was connected to the possibility to immerse or hide in it, for instance a shrub or high grass, or to offering objects (leaves and sticks) that can be used to transform the space into a place of imagination for sociodramatic play.

Reciprocal translation led us to conclude that when children played in nature-based environments, specific aspects of the nature-based environment, such as the abundance of materials and substances to play with might be connected to quality of children’s play activities, which is related to the cognitive outcomes mentioned above. At the same time the nature-based environment owns agency in play, “it/he/she plays back, nature instigates play.

#### Theme 3: Teacher-child interactions

In most of the studies in this review, children’s play in nature-based environments was child initiated, not teacher led. However, the role of the teacher is part of the children’s play environment and in four studies this teacher’s role in nature-based environment was specifically investigated (Mawson, 2014; Mackinder, 2017; Akpinar and Kandir, 2022). They found that the role of the teacher influences play quality. In the study of Mawson (2014) the outcomes of a hands-off approach to teacher child interactions, where children could freely roam throughout the woods, was compared to a hands-on approach with teacher-led activities. These two approaches resulted in differences in child behavior. In the hands-off approach, children were taking more risk and challenged themselves more and also engaged in more socio-dramatic play, while in the hands-on approach the teacher was directing children’s attention toward objects for play and shared more factual information.

It is important to also consider other factors that support possibilities of nature-based environments for children’s learning and development. Specifically, including assessments of teachers perceptions of their children’s underachievement, along with their supervisory/teacher style. In the study of Maynard et al. (2013), most of the children in the study that were perceived as “underachieving,” changed their behavior while playing in a nature-based environment to such extent that this “underachievement” was not seen anymore. To be outdoors in nature with more space and less constraining by teachers offered the children the opportunity to show differences in social, emotional, and learning behavior, for instance children were more cooperative, showed more pro-social behavior and remained more on task.

Reciprocal translation led us to conclude that when children play in nature-based environments, the character of the teachers’ mediation between children and between children and the environment influences how the affordances of the nature-based environment are actualized in play. When children received greater independent mobility license from their teachers (Kyttä, 2004) it not only offered more opportunities for risky play, but also for more independence in being creative, explorative, and self-confident. Moreover, teacher’s mediation itself is impacted by the nature-based environment: the nature-based environment changed their expectations of children’s skills and behavior, which in turn influenced children’s independent mobility license. The more affinity with the nature-based environment teachers had, the more they were able to reinforce children’s mobility and agency toward the nature-based environment, by balancing between child initiative and teacher initiative, transferring some of their own initiative to the nature-based environment.

## Discussion

Taken together our qualitative synthesis suggests that the affordances for play in nature-based environments experienced by children and teachers are not only different from the affordances for play in non-nature-based environments, which is obvious, but the affordances of the nature-based environment might also improve the quality of play. This is interesting for ECE teachers, since high quality play will yield children’s learning and development (Rubin et al., 1983). The studies also indicated that the relation between a nature-based environment and play quality is complex. Although the body of research into this topic is growing, more work needs to be done. The qualitative studies reviewed in this article forms a useful complement to the most recent systematic review on this topic from Dankiw et al. (2020), which reviewed primarily quantitative studies. Insights from the current review can support our understanding of the meaning of play that is enabled and sustained by the nature-based environment for children in ECE. Taken together, our review gives a first indication of the importance of play in nature-based environments for children’s cognitive, social-emotional, and motor development.

Qualitative research can thus unravel how children’s play and the nature-based environment are mutually constitutive and how play processes are mediated by teachers to support children’s cognitive, social-emotional, and motor development. Through an interpretation of the synthesis, below we present a “line of argument”–step 4 in the meta-ethnography–about how nature play can promote child development. We refine parts of play theory, by elaborating on the importance of the distinctive living character of the nature-based environment and its ability to “play back.” Besides, we will use the affordances theory to reframe the concept “afforded play actions.” We argue that reciprocity and diversity are unique qualities of nature play, contributing to child development if teachers permit and support children to explore the conceptual, social, technical, and metacognitive aspects of the nature-based environment in play.

### Line of argument, the value of nature play

Play theories explain how children’s active engagement with the surrounding world (i.e., play) results in knowledge of different aspects of the world, while in the meantime they learn to take part in it (Bakhurst, 2009; Piaget, 2013; van Oers, 2013). This qualitative synthesis illuminates the uniqueness of nature-based environments for meaningful play activity which is largely ignored in play theories Firstly the “living character” of the nature-based environment, the fact that it has a life of its own, accounts for reciprocity and diversity in children’s play. Secondly the fact that children use tools (or toys) during play is commonly accounted for in play theories, whereas nature-based environments provide an ample and diverse supply of loose parts (Speldewinde and Campbell, 2022). Which results in creative and imaginative play. Furthermore, both the stress reduction theory (SRT) as well as the ART account for the special connection between humans and nature-based environments (Ulrich, 1983; Kaplan, 1995; Adams and Savahl, 2017). These theories imply that being in nature contributes to wellbeing, but do not refer to interactions with nature. For children, being in an environment leads to interaction with it, and play theory shows that the quality of these play interactions is important (Burghardt, 2012; Speldewinde and Campbell, 2022). The current synthesis shows that, for children, not only *being* in nature but also *interacting* with nature is important, as they experience that these interactions are reciprocal. Nature has agency in these interactions and is adaptive toward diversity in children’s needs. Children listen to and tune into the nature-based environment, for example they gather sticks, pile them up for the imaginative bears to crunch them up during tea time. As such the environment instigates and enriches play.

In line with Gibson’s affordances theory, this review acknowledges how play actions are afforded by specific features of the physical environment and a specific user. However, we found that the affordances theory might overlook the complexity of the concept of “play” as it tends to look at individual play actions afforded by specific environmental features, such as a tree trunks affording jumping off. Using the affordances theory in this way, the attention will automatically be drawn to physical actions. Based on this qualitative synthesis, we argue that nature-based environments afford play activity on a more complex level than physical play actions alone. As we saw in the example of the children serving imaginative bears sticks during tea time, nature affords not only play actions, but also play scripts. The individual play actions are part of play activity that guides children to transform the perceptual world into a conceptual world. Our review indicates that nature-based environments afford the conditions for play, wellbeing, and involvement, as well as sociodramatic play and cognitive play, while in the meantime serving as a communicative context for sharing concepts together.

Our line of argument helps us to answer our research question: what is the value of play in nature-based environments compared to non-nature-based environments for developmental outcomes of young children (2–8 year). Our answer lays in defining how nature-based environments afford play in a distinctive way resulting in the concept of “nature play”: “play” in a nature-based environment consisting of natural loose and fixed elements (trees, vegetation water, sand, sticks, and stones) where children have the opportunity to engage in activities in which they are highly involved and where they have (some) freedom to develop their own play script, while interacting with and tuning into the affordances of the nature-based environment. Nature play has outcomes for cognitive, social-emotional, and cognitive development. In nature play, children have the possibility to find out how they are part of a **living** system. Early childhood educators are key actors in how children engage in play in the nature-based environment. They can support them to discover the conceptual, social, technical, and metacognitive aspects of nature-based environments. They need to expand children’s independent mobility to encourage them to explore the environment as well as to mediate between the child and the environment.

### Strengths and limitations

The strength of this systematic review is that it synthesized the meaning of play in nature-based environments in ECE across qualitative research. It is worth noting that although the synthesized studies were small-scale studies, these studies were particularly respectful to the way children interact with the world and sincerely tried to give voice to the view of these children and their teachers. Nevertheless, small scale studies are often context-specific lacking the scale to “follow through to the implied logical entailed conclusion” (Nye et al., 2016). Synthesizing the findings of these studies helps us to present new understandings of our topic, by drawing relationships between the individual studies. We acknowledge that the way we have refined and extended theory is not without its problems. A possible bias in the range and nature of qualitative research synthesized here is that outdoor play in ECE is mostly done for the reason of recess and to relax. For example, the strong emphasis on wellbeing and physical play in both the experiences of teachers and children, might reflect a western view on outdoor play in nature-based environments. Therefore, the reciprocal translation of the findings around cognitive skills were harder to synthesize although the importance of these findings for ECE should not be underestimated. Certainly, the strength of the meta-ethnographic approach is that it combines findings from multiple sources to increase validity and takes it a step further than primarily providing a narrative review of individual studies. Instead, it develops higher-order explanations. The consistency in the findings of studies in this meta ethnography supported its value, as the studies were undertaken in different educational settings, with nature-based environments varying in size and design. Another limitation is that in our attempt to translate themes across studies to arrive at higher order concepts during “step 2” of the synthesis, we may have lost some of the meaning and depth of key concepts and themes. However, we sought to preserve individual authors’ interpretations in our reciprocal translation of all the key concepts by memoing the key concepts. These memo’s contained comments on how the concepts were developed, connecting these concepts into meta themes, meanwhile we re-aligned our line of argument with the findings of the individual studies.

### Future research

This systematic review provides some suggestions for future research. The first promising line for new research would be to include a deep theoretical understanding of play for the development of young children when studying interventions in nature-based environments. Although the affordances theory seems to explain how the environments afford play actions, it is not sufficient to move beyond the individual play actions. From an educational perspective we argue it is important to shift our view of outdoor play from “letting off steam” to playing in nature-based environments for children’s cognitive development.

From a methodological perspective, future research could benefit from the post humanist view in the study of Harwood and Collier (2017). Taking the agency of the nature-based environment in the play of young children seriously, we might find new perspectives on how humans and nature are connected. This is in line with the movement of acknowledging the rights of nature, as was done for the first time with the Te Urewera Act in New Zealand (Parliamentary Counsel Office, n.d.). In this act, it is acknowledged that Te Urewera has an identity in and of itself, inspiring people to commit to its care. In a western view of nature-based environments we tend to look mostly at the human perspective of interaction with the nature-based environment, whereas in this synthesis it is clear that children experience nature as something that “plays back.”

### Conclusion

Results of this systematic review using a meta ethnographic approach indicates that playing in nature-based environments not only supports young children’s healthy physical development (e.g., physical activity and motor development), but might also support their social-emotional, motor, and cognitive development. Although the studies we reviewed were mainly explorative and small-scaled, they do indicate that nature-based environments have far more to offer than only a space to relax or let off steam. Nature-based environments function as a play partner that helps children to transform the perceptual world into a conceptual world, because it diversifies play, is sensory rich and it plays back. When playing in nature-based environments, children have the possibility to connect with it in an interactive way. When teachers know how to mediate children’s interactions with the nature-based environment, these interactions will have developmental value. Therefore, we encourage early childhood teachers to change their practice of playing outdoors into “nature play” as a daily activity that supports cognitive, social-emotional, as well as motor development. Finally, as we have seen the value of nature-based environments for play, in line with in Article 31 of the United Nations Convention on the Rights of the Child (United Nations, 1989) we might even consider nature play as a fundamental need and right of children. A need for and right to play in nature based environments that needs to be respected in the lives of young children.

## Data availability statement

The original contributions presented in this study are included in the article/Supplementary material, further inquiries can be directed to the corresponding author.

## Author contributions

All authors listed have made a substantial, direct, and intellectual contribution to the work, and approved it for publication.
