# Supplementary material for: Nature play in early childhood education: A systematic review and meta ethnography of qualitative research
Source: Front Psychol. 2022 Nov 10;13:995164. doi: 10.3389/fpsyg.2022.995164 (PMC9687100; doi:10.3389/fpsyg.2022.995164)
Supplement: Supplementary file 2 [file Data_Sheet_2.docx]

# Additional file 1 Search query and quality appraisal tool

## 1.1 Search query:

(AB =(green* NEAR/2 ((((((environment* OR playground*) OR landscape*) OR playscapes) OR setting) OR area) OR space*)))

 OR

(AB= (natur* NEAR/2 (((((environment* OR playground*) OR landscape*) OR playscapes) OR setting) OR area)))

OR

(AB= (school NEAR/2 ((((garden* OR forest*) OR wetland*) OR wilderness) OR grassland*)))

OR

(AB=(greenery))

OR

(AB= (garden*))

OR

(AB=(forest*))

OR

(AB= (wetland*))

OR

(AB=(wilderness))

OR

(AB= (grassland*))

OR

(AB= (tree cover))

OR

(AB = (tree canopy))

OR

(AB= (biodiverse school ground))

OR

(AB= (nature based))

AND

(AB= (“role play”))

OR

(AB=(play NEAR/2(((((pretend OR social) OR imaginative) OR "socio dramatic") OR "social pretend") OR "as if ")))

OR

(AB = (play NEAR/2 (((((((manipulat* OR object) OR relational) OR block) OR "loose part*") OR outdoor) OR free))))

OR

(AB = (play NEAR/2 ((((((((unstructur* OR "rough and tumble") OR explorat*) OR creativ*) OR construct*)OR physical) OR fysical) OR object) OR "gross motor")))

OR

(AB= (activit* NEAR/2 ((((physical OR unstructur*) OR explorat*) OR fysical OR construct *) OR "gross motor")))

NOT AB= ("plays a role”)

NOT AB=( “nature of”)

AND AB=child*

1.2 Quality appraisal tool for qualitative data (Lockwood et al., 2020)

Answers to the questions can be Yes(y) No (n) Unclear (u) or Not Applicable (na)

1. Is there congruity between the stated philosophical perspective and the research methodology?

2. Is there congruity between the research methodology and the research question or objectives?

3. Is there congruity between the research methodology and the methods used to collect data?

4. Is there congruity between the research methodology and the representation and analysis of data?

5. Is there congruity between the research methodology and the interpretation of results?

6. Is there a statement locating the researcher culturally or theoretically?

7. Is the influence of the researcher on the research, and vice- versa, addressed?

8. Are participants, and their voices, adequately represented?

Since the participants in these studies were very young children we appraised the studies that used creative ways to represent the actual voices of these young children with Y!, in our view this can be viewed as excellent.

9. Is the research ethical according to current criteria or, for recent studies, and is there evidence of ethical approval by an appropriate body?

10. Do the conclusions drawn in the research report flow from the analysis, or interpretation, of the data?

| Study | Q1 | Q2 | Q3 | Q4 | Q5 | Q6 | Q7 | Q8 | Q9 | Q10 |
| --- | --- | --- | --- | --- | --- | --- | --- | --- | --- | --- |
| 1.(Akpinar & Kandir, 2022) | u | y | y | y | y | y | n | y | y | y |
| 2. (Blanchet-Cohen & Elliot, 2011) | y | y | y | y | y | y | y | Y! | y | y |
| 3. (Herrington & Brussoni, 2015) | y | y | y | y | y | y | n | u | y | y |
| 4. (Canning, 2013) | y | y | y | y | y | y | y | Y! | y | y |
| 5. (Coates & Pimlott-Wilson, 2019) | y | y | y | y | y | y | y | Y! | y | y |
| 6. (Dyment & O’Connell, 2013) | u | y | y | y | y | y | y | Y! | y | y |
| 7. (Elliott, 2021) | y | y | y | y | y | y | n | y | y | y |
| 8. (Fjørtoft, 2001) | y | y | y | y | y | y | y | u | y | y |
| 9. (Harwood & Collier, 2017) | y | y | y | y | y | y | y | Y! | y | y |
| 10. (Lerstrup & Konijnendijk van den Bosch, 2017) | y | y | y | y | y | y | y | u | y | y |
| 11. (Luchs & Fikus, 2018) | y | y | y | y | y | y | y | u | y | y |
| 12. (Luchs & Fikus, 2013) | y | y | y | y | y | y | y | u | y | y |
| 13. (Mackinder, 2017) | y | y | y | y | y | y | y | y | y | y |
| 14. (Mårtensson et al., 2009) | y | y | y | y | y | y | n | n | y | y |
| 15. (Mawson, 2014) | y | y | y | y | y | y | y | y | y | y |
| 16. (Maynard et al., 2013) | y | y | y | y | y | y | y | y | y | y |
| 17. (Mcclain & Vandermaas-Peeler, 2015) | y | y | y | y | y | y | y | y | y | y |
| 18. (McCree et al., 2018) | u | y | y | y | y | y | n | Y! | y | y |
| 19. (Moore et al., 2019) | y | y | y | y | y | y | y | Y! | y | y |
| 20. (Morrissey et al., 2017) | y | y | y | y | y | y | y | y | y | y |
| 21. (Norðdahl & Einarsdóttir, 2015) | y | y | y | y | y | y | y | Y! | y | y |
| 22. (Puhakka et al., 2019) | y | y | y | y | y | y | n | y | y | y |
| 23. (Richardson & Murray, 2016) | y | y | y | y | y | y | y | y | y | y |
| 24. Sandseter, Ellen Beate Hansen | y | y | y | y | y | y | n | y | y | y |
| 25. (Storli & Hansen Sandseter, 2019) | y | y | y | y | y | y | n | u | y | y |
| 26. (Streelasky, 2019) | y | y | y | y | y | y | y | Y! | y | y |
| 27. (Wight et al., 2015) | y | y | y | y | y | y | y | y | y | y |
| 28. (Zamani, 2016) | y | y | y | y | y | y | y | Y! | y | y |
