# Supplementary material for: Nature play in early childhood education: A systematic review and meta ethnography of qualitative research
Source: Front Psychol. 2022 Nov 10;13:995164. doi: 10.3389/fpsyg.2022.995164 (PMC9687100; doi:10.3389/fpsyg.2022.995164)
Supplement: Supplementary file 3 [file Table_1.docx]

Table 1 Meta Method Extraction

| Study | Research Aim | Theoretical/philosophical perspective | Sample characteristics | Data collection | Data analysis | Results/Main Outcomes |
| --- | --- | --- | --- | --- | --- | --- |
| 1.(Akpinar& Kandir, 2022) | To investigate preschool teacher’s views on outdoor activities | Not specified | N = 63 preschool teachers (from all regions in Turkey). | Interviews, semi structured with 63 Turkish preschool teachers, on their views of outdoor play. | Thematic analysis of the interviews resulting in 9 themes. | 1. Teachers frequency of allocating time for outdoor play in their program  2. Teachers' views regarding reasons for not making time of making less time for outdoor activities in the program:  The weather is huge factor in the decision whether to go play outside or not. Not only teachers but parents as well are concerned about getting sick in cold weather.  3. Teachers' views regarding the time they allocate to outdoor play activities in the program.  4. Teachers' views regarding time preference for outdoor play activities.  5. Teachers' views on their planning styles of outdoor play activities in the program: Teachers would like to spend more time outdoors, Most of the teachers suggested that an improvement of the physical conditions of the playground, more clear educational policies on the benefits of outdoor play for children, and support of school management would make them spend more time in the outdoors.  6. Teachers' views on the ways of directing outdoor play activities in the program: They plan activities and have room for free play.  7. Teachers' views on the effect of outdoor play activities on development.  8. Teachers' views on the effect of outdoor play activities on other activities.  Most teachers in the study view outdoor play to be beneficient for social-emotional, physical and cognitive development. |
| 2. (Blanchet-Cohen & Elliot, 2011) | Describe how young children and educators actively engage outdoors | Right based, children’s right  Attention Restoration (ART) | N=12 (1-5 year), 4 early childhood centres specified N | Multisite Case Study with  participatory observation, videotapes, notes to measure children's play when playing outside. Focus groups with educators. | Thematic analysis of focus groups reflection process on incorporating natural environments as alternatives to usual playground structures. Reflection process with children and educators. | - eagerness, enthusiasm, joy, focus - involvement of senses, physical movement - including of natural elements in play - engagement with living creatures, - entertainment and discovery - learning about circularity, - cycles of more cooperative play, collaboration problem solving |
| 3. (Brussoni et al., 2017) | To investigate the effect of a Seven C's design: (character, context, connectivity, clarity, change, challenge) to increase access to nature and risky play opportunities  (highest quality play spaces: scaled to the child, sensitive to climate, include living materials, and elements that children can manipulate, and spaces for individual and group play | Affordances theory | N=45 children (2-5 year), 2 centres | Intervention study, repeated measures mixed methods design, measuring the change in play behaviour with: questionnaires sociometric status SDQ,  PSBS accelerometers play observations spatial behaviour maps ECE focus groups  Redesigning outdoor space by adding natural materials to enhance affordances for play | Comparative analysis T1 and T2 | Decrease in: depressed affect, antisocial behaviour and moderate to vigorous physical activity  Increase in: play with natural materials, independent play and prosocial behaviours |
| 4. (Canning, 2013) | To explore opportunities for creative thinking and imagination through den making, how do children use play space and resources to sustain imagination and creativity. | Cultural Historical Activity Theory (CHAT) | N=5 (3-4 year) | play observations, field notes on conversations of children, during den making sessions  Play observations on a woodland area | Thematic analysis | Using sticks for a ladder, using environment and the resources to keep it going, allowing children to go with their imagination, you can leave the materials where they are. Sustain the story, and creativity. Problem solving thinking through what they mean. |
| 5. (Coates & Pimlott-Wilson, 2019) | To investigate how children interpret their learning experiences while engaging in forest school setting | Constructivist, CHAT | N=33 (4-8 year), 2 schools | Semi structured interviews with children on their experience of classroom learning compared to outdoor engagement in Forest School to generate understanding about the meaning of these experiences.  Comparing classroom learning with forest school programme | Phenomenological thematic analysis | - break from routine - no pressure, no stress, refreshed, playful - learning through play - being creative - kinaesthetic , experiential learning, making things, story-based learning, child   directed play in FS   - being physically active - learning about the environment, navigate challenging environment, - managed risk - collaboration and teamwork - the opportunity to experience a change in social boundaries |
| 6. (Dyment & O’Connell, 2013) | To investigate if play design influences where and how children play on preschool playgrounds | Not specified | N=100, (1-5 year) 4 schools, 4 playgrounds | Observation of play of play on differently designed target areas of different designed outdoor spaces: paths, paved places, manufactured equipment, soft falls, natural areas, sand pits using scans with SOPLAY | Comparative analysis | Paved area's: mostly functional play, chosen when there’s is no alternative Softfall and grass : functional play, self-focused: popular choice Sand features: for constructive and symbolic play, chosen if there is no natural alternative Manufactured, constructions: popular choice for symbolic and functional play depending on type of equipment Natural area's: even if small popular choice functional and constructive play that was creative an imaginative |
| 7. (Elliott, 2021) | To examine the ways in which children use the garden in different seasons and to discuss factors, such as the weather and the adults in the setting, that affect their play alongside the affordance of outdoor resources available to them. | Reilly’s play theory  Affordances theory | N=50, early childhood setting with a garden | Observation of play of 50 children, during observation field notes were made, with notes on the weather circumstances. Also pictures were made of play activities. | Thematic analysis, with focus group and children | There is a strong connection between the resources available to children in their play, the other players (including adults and their role in the play) and the  seasonal changes in the weather. Children needed more encouragement of adults when it was cold. But when outside they modify and adapt resources according to their needs. Having a choice, makes children to spend more time outdoors. Adults are likely to make choices according to the weather circumstances, while children choose to go outside anyways, and adapt their play to the circumstances. The nature and space of the outdoor environment makes it possible to practice skills in a different way than indoors. |
| 8. (Fjørtoft, 2001) | To investigate how children's play in the natural environment might stimulate their motor fitness, by focusing on the affordances of the landscape and the correlation with versatile play | Affordances theory | N=75 | Measuring children's motor fitness after a nine months period of play on two different outdoor environments: a forest and a fenced outdoor space. Pre- and post-test with EUROFIT test for motor fitness.  Comparing play in forest nearby kindergarten with play on traditional playground | Multiple regression analysis | Structures and affordances for play and the impact on motor development in children. Significant relationship between diversity of the landscape and the affordance of play. Landscape might have a functional impact on children's play behaviour. This impacted the motor fitness. Pre-test and post-test improvement on all elements in the experimental group. Balance an coordination improved significantly. |
| 9. (Harwood & Collier, 2017) | To observe and document intra-active and improvisational entanglements with the forest as a more than human world. | Post humanist | N=8 (2-4year) | Thing-matter-energy-child assemblages, while playing in forest during Forest School sessions. Data collected with: note-books, iPads, Go-Pro camera's  Exploring play in forest school site | Thematic analysis, post humanist theorizing | The sticks were  1. non-representational aspects of children's play, substitutions for the real thing 2. embodied, entangled within the children's play, beyond representation 3. agents, the agency becomes entangled within the agency of the agency of the children4 4. Changed the dynamics of the play with their presence 5. vibrant an actant 6. entangled with the child, child an d stick are vial tot the production of something created  Independent role of nature, agency of sticks |
| 10. (Lerstrup & Konijnendijk van den Bosch, 2017) | aim: a practical way to describe and classify outdoor settings for children's self-initiated activities in preschool, with affordances as the synthesising concept which terms are appropriate for analysing and understanding affordances of outdoor settings for children in preschool | Affordances theory | N=49, 2 schools traditional playground, forest play space | observation of meaningful activities and environmental features that seemed to be of value during children's play in two different outdoor environments: a forest and a traditional playground, using video recordings and field notes.  Comparing traditional playground with forest nearby preschool | Comparative analysis of activities that seemed ‘no matter’ and features that seemed to be of value | 5 affording features, 8 functional affordances, places: Open ground, sloping terrain, shielded places: run drive walk/ roll slide, clamber/ hide as a frame attached objects, Rigid or moving fixtures: climb balance, jump/ swing sway, seesaw, spin detached objects, loose objects: Arrange, modify as tools, props, treasures substances, loose materials, water: dig, move, mould, smear/pour mix, splash, float ( new) events, creatures and fire: look for, handle care/ feed, look after, sit by |
| 11. (Luchs & Fikus, 2018) | Revealing the impact of different playground environments on the locomotive activity, the range of activity levels during free play. To consider if kindergarten environments should be more diverse, incorporating both elements and structures of natural and contemporary playgrounds | Affordances theory | N=17 (5-7year) | measures of children's locomotive activity in two different designed playgrounds: a natural playground and a traditional playground, using pedometers.  Comparing contemporary playground with park | Comparative analysis | gait cycles pm: natural: 25, SD=4.99, min:16.59,max:35,41 contemporary: 28,55. SD=9,60, min 6,82, max:51,00 More gait cycles in contemporary, but also greater SD. Distribution of differences during the whole play episode is different between natural and contemporary. In natural play area not only active children are activated, but also less active children find opportunities to be active. Natural environment |
| 12. (Luchs & Fikus, 2013) | To explore the impact of different designed playgrounds on the play behaviour of children a. detailing number of play episodes b. occurrence of the different categories of play c. duration of the play episodes d. patterns of the play episodes. | Play theory (Frost)  Affordance theory  Loose parts | N=59 (5-6year) | Observation of two different designed playgrounds: a natural playground and a traditional playground. Measures: Number of play episodes Occurrence of different play categories play with, play as, play for, Duration of play episodes Patterns of play episodes.  Comparing contemporary playground with park | Comparative analysis | Naturally structured playground: play episodes are longer, sometimes 1 episode for 30 minutes. Suggesting a high degree of concentration, less play with and play for, more combination patterns: more combination of categories. Contemporary playground: episodes are never longer than 15 minutes. |
| 13. (Mackinder, 2017) | To explore the involvement of children and the participation of adults in Forest School sessions alongside investigation of how sessions are planned. | Forest School approach | N=1 case study 1 child and two teachers | Observations, field notes, audio recordings of children's play of two forest school sessions with two different ECE teachers using Leuven Child Involvement Scale and Adult participation Scale for adults  Exploring movements on forest school site | Comparative analysis of teacher style on child behaviour | Eve scores higher on autonomy, sensitivity and stimulation. Eve was full of energy motivation positive, full of praise, caring, allowing for the child's experimentation and exploration. Gill was caring and affectionate but it was coupled with dominating and authoritative behaviour. In the interview, Eve talked about deep learning, and Gill more about boundaries. planning styles: initially it seemed that there was difference in planning style, but in practice it appeared to be both adult led. Tracking map showed how the child explored more autonomously in Eve's session. Eve was more trained in forest school practice than Gill was. ( training amplifies the added value of nature, mobility licence is greater) |
| 14. (Mårtensson et al., 2009) | To investigate  if the attention of preschool children is related to outdoor environments, with different play potentials, assesses by OPEC score.  If the attention of preschool children is related to outdoor environment with different proximity between natural elements and play structures, assessed by sky view factor. | Attention Restoration (ART) | N=198 (4-6 year), | Observations of children's inattententive, hyperactive and impulsive behaviour using ECCADES tool in different outdoor play environments differences assessed with OPEC  Comparing differently designed playgrounds, assessed with ECCADES tool | Statistical analysis,  correlation between OPEC score and ECCADES outcomes | High OPEC score relates significantly with inattention, close to significant with hyperactivity and impulsivity. (these factors were also impacted by mother’s education, children’s outdoor time on Sundays and outdoor fraction, and if child is content with preschool) No relation with sky view factor. Long outdoor stay seemed to be negatively related to attention, but only for preschools that were outdoors all day. |
| 15. (Mawson, 2014) | To explore how teachers views and interaction with the wildwoods influences the way children interact with it | Affordances theory  Theory of place (Tovey) | N=16 | Observation of teacher-child interactions during children's free play within a wilderness outdoor setting, field notes, interviews with teachers, and photographs.  Exploring interactions in a wooded area | Thematic analysis | Environment contained variety in affordances with multiple play opportunities. Teacher interactions could be placed on an continuum that leaved children to freely roam throughout the woods, just checking on their safety, to very teacher-lead activities. Hands-on approach: direct attention to objects, more factual information, which was used afterwards to be able to do it their selves afterwards ( finding the right marshmallow sticks) more complex activities Hands-off approach: more opportunities for taking risks and physically challenging themselves and to draw the teacher's attention. More physically challenging play and more sociodramatic play, but the themes were not shaped by the environment. (mobility licence and guidance) |
| 16. (Maynard et al., 2013) | to find out whether child- initiated learning outdoors did have any effect on children who were perceived by their teachers to be 'underachieving ‘and if so, the extent nature and possible reason for this effect. | Reggio Emilia Approach, child initiated learning | N=48, (4-7year) 8 teachers, | interviews with teachers, field notes of key issues and insights, reflective journals, final project report of participants on their view of children's learning competence during child initiated outdoor play.  Comparing green outdoor spaces with indoor settings | Comparative thematic analysis | 28 of the children acted better in child-led activities. 10 in the social and 9 in the emotional 9 in learning domain 17 children about whom teachers did not make any specific judgements in their final project reports. They tended to comment more generally on observed differences 3 children did not show any positive difference in behaviour, they had multiple severe difficulties, either developmental or challenging home circumstances reasons for observed differences:  To be outdoors calm some children down, where there is more space and less constraint by teachers Child initiated learning Changing perceptions of underachievement |
| 17. (Mcclain & Vandermaas-Peeler, 2015) | To explore in which ways two natural environments influence pre-schooler’s physical and socioemotional development, including ways they interacted with various environmental affordances and their peers | Affordances theory | N=11(2-5year) | Observations of children's play in two different outdoor environments; a creek and a river, using videotapes  Comparing two nature based environments that differed in ‘wildness’ | Comparative analysis two environments | Creek: flat surfaces: 27%, mostly afforded running, hiding, balancing, jumping off  River: 16 % mostly afforded running and hiding, but also balancing, climbing, jumping. Emotions at creek and river mostly neutral/positive. Personal challenges: River more challenges than creek, mostly in climbable or water affordances Relation between affordance and physical and play behaviour, the wilder environment(river) afforded more risk which results in the development of confidence in the face of risk |
| 18. (McCree et al., 2018) | To explore the impact of forest school attending on academic attainment, wellbeing and connection to nature. What are the significant changes over the longitudinal span of the project? | Not specified | N=11 (5-7 year  Longitudinal 3 years | Wellbeing involvement and engagement measured with Leuven involvements scale. Connection to nature measured with Connection to nature index. Academic attainment and attendance measured with teacher's assessments of reading writing and maths. Child interviews, case studies,  questionnaire’s for staff and parents to find themes.  Comparing attending forest school programme with children that did not attend the programme | Comparative thematic analysis | Wellbeing, involvement and engagement: high scores, over three years Nature connection: higher in study group compared to average in national survey Academic attainment and attendance: reading, writing and maths, higher improvement than control group |
| 19. (Moore et al., 2019) | to explore children's perspectives on and states of wellbeing while playing, revealed in their story telling, while playing in two different outdoor play environments | Theory of place (Grünewald)  Play theory (Huizinga) | N=30 ( 4-5year) | mosaic approach in two centres with different outdoor environments: one with manmade equipment, limited natural surfaces and vegetation one with a playground containing more natural elements and also access to a community garden:  Drawings of elements in outdoor play space, Direct tour and photograph important elements Collections of artefacts from outdoor play space into 'memory box' Creating maps of important outdoor play spaces using collected artefacts and photo's Position wishing stones in particular space and re-imagine that space  Comparing differently designed outdoor spaces | Comparative thematic analysis | Stories of agency, stories of place and attachment, stories of hiding Agency: wellbeing includes the notion of self-confidence and a sense of feeling capable. Relation between positive outcomes and combination of traditional playground equipment with natural elements. Widespread areas of natural shade, provided by trees. Not crowded. Abundance of loose materials, lot of secluded areas. |
| 20. (Morrissey et al., 2017) | To explore the potential influence of the nature of the outdoor play space and associated resources on children's enactment of sociodramatic play processes, | Cultural Historical Activity Theory (CHAT)  Affordances theory | N=28 (4-5year) | Event sampling of play episodes in two different designed outdoor environments: yard 1: a traditional space with manmade equipment and a naturalised play space defined by natural features such as shrubs, logs, rocks, plants.  Comparing a traditional outdoor space with a highly naturalized one | Comparative analysis | More sociodramatic play in natural yard in minutes, episodes persisted longer. With lesser children, children spent more time in natural yard. Children in natural yard were less likely to confine their play episodes to one or two areas. In natural yard sociodramatic play was more likely to integrate physical movement. Fantasy roles more likely in natural yard and more domestic roles in traditional yard. |
| 21. (Norðdahl & Einarsdóttir, 2015) | To explore children’s preferences about outdoor activities and surroundings in the outdoor school environment | Affordances theory | N=8 (4-5 year) N=8(7-9 year) | interviews, walking tours conversations with children,  meetings with teachers, classroom observations  Comparing differently designed playgrounds | Thematic analysis | - Physical challenge - Explore things - Be in contact with others - Enjoy beautiful things |
| 22. (Puhakka et al., 2019) | To explore how simultaneously increasing biodiversity exposure and greening yards, is perceived to affect 3-5 year-old children's physical activity and play, their environmental relationships, and their wellbeing in the urban environment in Finland. | Affordances theory | N=? | Interviews with 12 daycare givers and a survey with the 12 daycare givers and 49 parents on possible changes that took place after greening of the yards. | Qualitative content analysis | Functional affordances: Physical activity, multi-sensory experiences, diverse play, arts and crafts, nature exploration, pre-academic skills.  Embodied experiences: increase in physical activity and diversification of activity.  Involvement: more creative play and imaginative role-playing. They also were looking after the plants and vegetation.  Exploration: Increase in self-guided exploration and exploratory play. |
| 23. (Richardson & Murray, 2016) | To explore if there are links between the environment and the quality of young children's utterances as part of their speech and language development. | Theory of language construction, Tomasello | N=4 (3-5year) | Multiple case study, measuring language reasoning element with TTR (type token ratio) in two different outdoor environement rated with the ECERS scale: the outdoor classroom and a forest school site.  Comparing traditional outdoor classroom with forest school site | Qualitative content analysis, Type-Token  Ratio analysis | TTR: verb use higher in the natural environment for all children. Exclamation usage and adjective usage was higher for three of four children. Noun usage was higher in classroom for two children compared to forest school. Thematic analysis: semantics were highly connected with the environment. ECERS was lower in natural setting but diversity in language was higher. increased action focus in natural environment might elicit more verb use. More adjective onomatopoeic, connected to more sensory richness. More physicality of the natural environment might promote children's communication. More exclamation, greater freedom to express themselves.  lesser diversity in noun use can be related to a higher involvement in one activity with a limited number of objects, not flitting between experiences and encountering al lot of objects. |
| 24. Sandseter, Ellen Beate Hansen | To explore qualitatively the affordances for risky play of two environments, the potential of both playgrounds will be evaluated for children's mobility license and actualized affordances, how different environmental features afford risky play and how these affordances are actualized in children's play. | Affordances theory | N=29 (4-5year) | Observation of play behaviour in two different outdoor environments: an ordinary 'fixed' playground and a forest. Interviews with the children on categories of risky play: play with:  Heights, Speed, Dangerous tools, Dangerous elements, Rough and tumble play, Dissappear/get lost.  Comparing traditional playground and nature preschool site | Content analysis using potential and actualized affordances | Ordinary preschool: potential affordances, actualized affordances: great heights and great speed mobility licence: extensive Natural preschool: potential affordances and actualized affordances: great heights, great speed, mobility license: extensive  Difference: disappear/ get lost only in nature. Nature afforded risky play of higher degree of risk |
| 25. (Storli & Løge Hagen, 2010) | To find out how two different play environments influence activity levels of preschool children | Affordances theory | N= 16 (3-5 year) | Play observations in two different play environments: the preschool's playground in winter and spring, and a nature excursion in spring. Observation of main group activities, related to different environment features and individual activities related to physically active play, which was also measured by accelerometers  Comparing traditional playground with natural playground | Comparative statistical analysis. Analysis of actualized affordances | Children have the same activity levels in both play environments. standard deviation was less in the measurements of the natural environment. strong correlation physical activity level and individual features  The lowest activity levels were measured in the traditional playground, it seemed that there was more boredom in the traditional playground for the older children. influence of environmental conditions that change that transform environmental features temporarily |
| 26. (Streelasky, 2019) | To explore what learning experiences do children value at school? What modes are they choosing to express and represent their valued school learning experiences  Exploring play in a forest | Play narrative theory (Bhaktin) | N= 15 (5-6 year) | Play observations during play in the indoor classroom setting and a large forested area close to the school. 2 semi-structured interviews with every student. Multi model participation: photography, painting and drawing and in writing in journals and reading levelled books.  Comparing traditional playground with natural playground | Narrative analysis, image-based analysis, thematic content analysis | Majority of the children shared stories about the value they placed on their outdoor experiences. They valued being outside with their peers and with nature. The narratives revealed that children conceptualise themselves there as social beings, and the data provided some insight into the strong sense of autonomy they felt when they were engaged in collaborative outdoor play. |
| 27. (Wight et al., 2015) | To investigate what the difference is between pre-schoolers playing on a traditional playground and in nature when it comes to fostering inquiry and exploration as a prerequisite to environmentally responsible behaviours. | Play theory (Piaget) | N=64 (3-5 year) | Play observations of three visits to a playscape: a large fenced area, containing large amounts of natural elements such as a forest, wetlands, stream, rock formation and more, using videotapes.  Comparing traditional playground with playscape | Thematic analysis using codes for levels of inquiry: observation, exploration, representing and recording, language, functional play, constructive play, dramatic play, games | Playscape: Area of Interest (AOI) 42,7% water and 33,6% woods functional play:61,8% constructive play: 26,4 dramatic play 11,8% Science inquiry: observation 34,5%, exploration 43% representation: 5,2%, science specific language: 17,3 % ( naming plants and animals and life concepts) Playground AOI: sandpit, activity area 1(corn shucking) and 2: bubble blowing station and bike track. sandpit: 50,3%, activity 1: 40,7%, activity 2:3,8%  play behaviour: functional 42,4 %,constructive: 55,2% dramatic: 2,3 % Inquiry related: observations: 39,6 % explorations 46,3% science specific language: 14% More diversity in AOI playscape, More science inquiry( specially representing and language in playscape, playscape fostered more Environmental responsible behaviours |
| 28. (Zamani, 2016) | To explore how children and early childhood teachers view the cognitive play opportunities in outdoor preschool ones with different proportions of natural settings, how can you compare these settings? | Play theory (Rubin) | N=58 (4-5year) | Children made drawings and choose photographs of places where they liked to play. Than they were interviewed to explain their preferences. Interviews with teachers on outdoor spaces as a learning environment. Behaviour mapping observations  Comparing play on natural, mixed and manufactured and zones of an outdoor preschool | Thematic content analysis | Children: preference for functional play, explorative play in nature, dramatic play, and games in the natural zone, loose parts for cognitive play. Teachers: value of outdoor for learning, learning capacity for of natural zones. Outdoor space is an extension of indoor curriculum to develop social physical and cognitive experience. Each zone has different capacity to support a certain part of development. Nature area offered higher levels of cognitive play, twice as much constructive and the most exploratory and dramatic play. Loose and maliabe parts supported these types of play. Exploratory experiences stimulate children's scientific curiosity to create realistic theories about the world. |
